# The nature, consequences, mechanisms, and management of sleep disturbances in individuals at-risk for psychosis

**DOI:** 10.3389/fpsyt.2022.1011963

**Published:** 2022-09-20

**Authors:** Feten Fekih-Romdhane, Souheil Hallit, Majda Cheour, Haitham Jahrami

**Affiliations:** ^1^Faculty of Medicine of Tunis, Tunis El Manar University, Tunis, Tunisia; ^2^The Tunisian Center of Early Intervention in Psychosis, Department of Psychiatry Ibn Omrane, Razi Hospital, Tunis, Tunisia; ^3^School of Medicine and Medical Sciences, Holy Spirit University of Kaslik, Jounieh, Lebanon; ^4^Psychology Department, College of Humanities, Effat University, Jeddah, Saudi Arabia; ^5^Research Department, Psychiatric Hospital of the Cross, Jal Eddib, Lebanon; ^6^College of Medicine and Medical Sciences, Arabian Gulf University, Manama, Bahrain; ^7^Department of Psychiatry, Ministry of Health, Manama, Bahrain

**Keywords:** sleep, UHR, at-risk mental state, psychosis, early intervention

## Abstract

There is strong evidence that sleep disturbances are commonly experienced by people with psychosis. Evidence has also shown that sleep disturbances are present since the very early stages of the disease, even during the pre-diagnostic phase. More recently, research involving young individuals at ultra-high risk (UHR) for psychosis documented frequent occurrence of sleep disturbances in this group. The very early onset of sleep disturbances in the course of psychosis has drawn attention to the possible links between sleep parameters and the risk of psychosis. To date, the nature of sleep disturbances characterizing the UHR stage remains unclear, with available studies having yielded mixed findings. In this regard, we performed this review to update the body of literature on the nature of sleep disturbances, their underlying mechanisms, their clinical and functional consequences, the prevention and intervention strategies in the at-risk for psychosis population. Our findings provided further support to the presence of disturbed sleep in UHR individuals as evidenced by subjective and objective sleep measures such as polysomnography, sleep electroencephalograms, and actigraphy. Reviewing the possible mechanisms underlying the relationship between sleep and psychosis emphasized its complex and multifactorial nature which is yet to be determined and understood. Further research is warranted to determine which facets of sleep disturbances are most detrimental to this specific population, and to what extent they can be causal factors or markers of psychosis.

## Introduction

Sleep is an essential biological function that results from a complex interaction between neurobiological, hormonal, and homeostatic processes. Sleep disturbances are highly prevalent worldwide, affecting one out of five adult people in the community (1). In particular, there is strong evidence that sleep disturbances are experienced to a higher extent by people with psychosis than healthy individuals (2, 3). Substantial sleep problems are reported by up to 80% patients with psychosis in remission (2, 4), are linked to impaired cognitive and functional capacities (5), and contribute to a significant decrease in life expectancy (6). Evidence has also shown that sleep disturbances are present since the very early stages of the disease (7), even during the pre-diagnostic phase (8, 9). More recently, research involving young individuals at ultra-high risk (UHR) for psychosis documented frequent occurrence of sleep disturbances in this group (10, 11). The very early onset of sleep disturbances in the course of psychosis has drawn attention to the possible links between sleep parameters and the risk of psychosis; and has led some authors to suggest that sleep problems are not causally related to chronic symptoms or medication but rather appear to be promising biomarkers of the disease (7, 12). The UHR state refers to the presence of one of the following: attenuated psychotic symptoms, brief limited intermittent psychotic episode, and genetic risk and deterioration syndrome (i.e., trait vulnerability with marked decline in functioning) (13). Being able to intervene during the UHR stage through targeting modifiable factors, such as sleep, offers the opportunity to be more impactful on outcomes by resorting to simpler, more personalized and less harmful treatments (14–16). However, the nature of sleep disturbances characterizing the UHR stage remains unclear, with available studies having yielded mixed findings (10). In addition, potential mechanisms involved in the association between sleep and psychosis etiology are still largely unknown (17). We could find only one previous review that included data prior to February 2020 to examine sleep disturbances in the UHR state, with the specific goal of exploring the relationships between sleep and psychotic symptoms, functioning and quality of life (18). Some interesting new studies have emerged since then [e.g., (19–22)]. This, along with the identified knowledge gaps, have motivated the present review aiming at synthetizing the existing literature to update and extend our understanding of: (1) the nature of sleep disturbances, (2) their underlying mechanisms, (3) their clinical and functional consequences, as well as (4) the prevention and intervention strategies in the at-risk for psychosis population.

## Methods

In the present mini-review, we performed a literature search using electronic databases (i.e., Pub Med, Web of Science, and Scopus). All studies have been selected according to the following criteria: (1) original peer-reviewed articles written in English, with no time period limits; (2) study samples comprising individuals (aged 12–35 years old) defined as meeting UHR criteria based on the presence of attenuated psychotic symptoms, genetic risk, and functional deterioration; as ascertained by structured clinical interviews [e.g., the Comprehensive Assessment of the At Risk Mental State (CAARMS) (23); the Structured Interview for Prodromal Symptoms (SIPS) (24), the Structured Clinical Interview for DSM Disorders (SCID) (25)]; and (3) studies providing data on sleep disturbances in UHR individuals as assessed using either subjective or objective measures. The articles were selected using cross-matched keywords combination: [At-Risk Mental State [OR] Clinical high risk state [OR] Ultra-high risk [OR] prodromal state [OR] prepsychotic phase [OR] psychosis risk [OR] emergent psychosis [OR] early psychosis) [AND] (sleep [OR] sleep disorders [OR] sleep problems [OR] sleep disturbances [OR] sleep quality [OR] insomnia [OR] sleepiness [OR] circadian rhythm [OR] chronotype [OR] polysomnography [OR] actigraphy]. In addition, a backward search and a Google search were conducted in order to detect any other possible missing relevant research studies or unpublished (gray) literature.

## The nature of sleep disturbances in UHR individuals

There are two ways to measure sleep disturbances; one is subjective through self-reported [e.g., PSQI (26)] or interviewer-reported [e.g., Scale of Prodromal Symptoms (SOPS)/Structured Interview for Psychosis-Risk Syndromes (SIPS) sleep disturbance items (24)] sleep questionnaires, and the other is objective (e.g., sleep parameters/architecture according to actigraphy or polysomnography). As such, this section will be divided into two parts; the first part reports findings on subjective sleep disturbances, while the second one includes characteristics of objective sleep assessments for UHR individuals (see Table 1 for further details on key findings of the reviewed studies).

**Table 1:** Summary of the previous research findings on sleep disturbances in UHR individuals^*^.

**First authors**	**Country**	**Study design**	**Study population**	**Sleep measures**	**Summary of key findings**
**Studies using subjective measures of sleep**					
Goines (31)	Canada, USA	Longitudinal	*N* = 740 UHR (43% females; aged 18.5 ± 4.26 years) + 280 HC	SOPS sleep disturbance score	• UHR individuals reported higher levels of sleep disturbance than HC (mean sleep disturbance scores of 2.31 ± 1.568 vs. 0.48 ± 0.904, respectively). • In the UHR group: - Baseline sleep disturbances were significantly linked to greater positive symptoms (i.e., paranoia and hallucinations). - No significant differences were found in baseline sleep disturbance between participants who remitted, remained symptomatic, had prodromal progression, or converted to threshold psychosis during follow-up.
Grivel (27)	USA	Longitudinal	*N* = 200 UHR (28% females)	SIPS sleep disturbance score	• UHR individuals with any lifetime trauma (*n* = 47) had significantly higher sleep disturbance than those with no history of trauma (*N* = 153) (mean sleep disturbance scores of 3.17 ± 1.539 vs. 2.50 ± 1.727, respectively)
Lederman (34)	Australia	Cross-sectional	*N* = 10 UHR (20% females) + 10 FEP + 10 HC	PSQI	• UHR participants had significantly poorer overall sleep quality than FEP patients and HC (PSQI total scores of 8.0 ± 3.3, 5.5 ± 3.4 and 3.9 ± 1.5, respectively; *p* = 0.01). Specifically, daytime dysfunction and sleep medication use (psychotropics prescribed with the primary purpose to improve sleep) were significantly greater among UHR participants than both FEP patients and HC.
Lindgren (40)	Finland	Longitudinal	*N* = 54 UHR (81% females; aged 16.7 ± 0.85 years) + 107 non-UHR psychiatric patients	SIPS sleep disturbance score	• Sleep disturbance was significantly associated with current and lifetime suicidality. No association was found between sleep disturbance and intentional self-harm during follow-up (mean sleep disturbance scores of 2.5 ± 1.4 in “No self-harm” group as compared to 2.0 ± 1.2 in “Self-harm” group, *p* = 0.43).
Lunsford-Avery (12)	USA	Cross-sectional	*N* = 33 UHR (33% females; aged 18.73 ± 1.89 years) + 33 HC	PSQI	• UHR adolescents had significantly higher PSQI total scores, increased latency and greater disturbances compared to HC. • In the UHR group: - More sleep difficulties (increased latency, reduced quality and duration of sleep) were significantly associated with increased negative symptoms. - No association has been found between PSQI variables and positive symptoms. - Bilateral thalamus volume reductions were linked to increased latency, reduced efficiency, and decreased quality of sleep.
Lunsford-Avery (41)	USA	Cross-sectional	*N* = 59 UHR (42% females; aged 18.93 ± 1.67)	PSQI	• A total of 23 UHR participants (33.9%) had poor sleep quality (PSQI>8) • “Poorer sleepers” exhibited lower overall cognitive performance, increased negative symptom severity and similar functioning levels compared to “better sleepers.” • Sleep disturbances (i.e., latency, efficiency and sleep quality) were significantly associated with procedural learning deficits.
Michels (36)	Germany	Cross-sectional	*N* = 14 UHR (36% females; aged 23.29 ± 3.91 years) + 17 patients with schizophrenia + 17 Healthy relatives of patients with schizophrenia + 29 HC	Self-developed Likert-type single items assessing self-reported frequency of dream recall and nightmare during the last 2 months	• UHR participants reported higher nightmare frequencies compared to patients with schizophrenia, first-degree relatives and HC (Means of nightmare frequency of 3.79 ± 1.93, 3.65 ± 2.50, 2.41 ± 2.00, and 1.90 ± 1.92, respectively). • UHR participants had higher dream recall frequencies compared to patients with schizophrenia (while relatives and HC reported lower and similar mean scores)
Miller (28)	Canada, USA	RCT	*N* = 60 UHR (35% females; aged 17.8 ± 4.8 years)	SOPS sleep disturbance score	• Sleep disturbance was reported by 37% of UHR participants (which represents the percent of patients scoring between 3 “moderate” and 6 “extreme” in the SOPS sleep item)
Nuzum (19)	UK	Retrospective	*N* = 795 UHR (44% females; aged 22.72 ± 4.89)	Sleep disturbances reported by clinicians (Any form of insomnia or disturbed sleep that happened more than once and was having an impact on the client's life)	• 59.5%, of UHR individuals experienced sleep problems (22.01% and 58.11% of individuals reported insomnia and disturbed sleep, respectively)
Poe (30)	USA	Longitudinal	*N* = 194 UHR (27% females; aged 20.0 ± 3.8 years) + 66 HC	SIPS sleep disturbance score	• UHR subjects displayed significantly higher sleep disturbance scores than HC. • In the UHR group, sleep disturbance was related to higher positive and negative symptoms and more impaired functioning.
Reeve (50)	UK	RCT	*N* = 160 UHR (39% females; aged 20.9 ± 4.2 years)	Economic Patient Questionnaire Interview	• At baseline, 85% of UHR individuals experienced ‘Bad' night with a mean sleep duration of 4.14 h. • The baseline cross-sectional evaluation revealed that a shorter sleep duration was significantly associated with increased positive symptoms (delusional ideas and hallucinations) and distress.
Ruhrmann (39)	England Finland Germany Netherland	Longitudinal	*N* = 245 UHR (44% females; aged 23.0 ± 5.2 years)	SIPS sleep disturbance score	• Sleep disturbances score >2 on SIPS helped predict transition to psychosis at 18-month follow-up.
Tso (29)	USA	Cross-sectional	*N* = 203 UHR (43% females; aged 16.8 ± 3.3 years) + 87 individuals with clinically lower risk + 44 very early FEP (<30 days of positive symptoms)	SOPS sleep disturbance score	• UHR participants displayed higher sleep disturbance scores than individuals with clinically lower risk (Scores of sleep disturbance were the highest in FEP patients).
Waite (90)	UK	Qualitative	*N* = 11 UHR (54% females; aged 18.27 ± 1.95 years)	Interviews	• Participants reported delayed sleep phase, lack of routine, circadian rhythm disruption (i.e., day-night reversal). • They also described a complex and reciprocal relationship between sleep disturbance, mental health problems, and daily functioning.
Zaks (20)	USA	Longitudinal	*N* = 478 UHR participants: 67 converters to psychosis (46% females, aged 18.85 ± 4.02 years) and 411 non-converters (45% females; aged 18.30 ± 4.10 years) + 94 HC	PSQI RU-SATED questionnaire	• All PSQI subscores (i.e., duration, latency, disturbance, efficiency, daytime dysfunction, subjective quality, and medication use) and total score were significantly higher in UHR participants (at a similar extent between converters and non-converters) related to HC; indicating an overall poor sleep quality in UHR groups compared to good sleep quality in HC. • No significant differences were found between UHR and HC individuals in napping frequency or the RU-SATED items timing and regularity. • In UHR individuals, baseline disturbed sleep did not predict conversion to psychosis up to >2 years later. • Sleep disturbance was strongly associated with increased positive symptoms over time.
**Studies using objective** **±subjective measures of sleep**					
Castro (33)	Brazil	Cross-sectional	*N* = 20 at-risk individuals: 13 UHR and 7 at bipolar risk (35% females; aged 18.3 ± 4.01 years) + 20 HC	Actigraphy PSQI ESS MEQ	• Participants of the at-risk group had worse sleep quality compared with HC (PSQI total scores of 7.70 ± 3.69 compared to 4.95 ± 2.16, respectively; *p* = 0.010); whereas no significant differences were noted between the groups regarding sleepiness and chronotype profiles. • The actigraphy data indicated that the at-risk group displayed longer nap duration during waking (44 vs. 23 min), lower autocorrelation functions, lower interdaily stability, higher intradaily variability, lower most active 10 h of the day (M10), and higher beginning of the M10.
Lunsford-Avery (35)	USA	Longitudinal	*N* = 36 UHR (47% females; aged 18.73 ± 1.89 years) + 31 HC	Actigraphy PSQI	• The actigraphy data revealed that UHR participants presented increased WASO, decreased efficiency, and increased movements during sleep relative to HC • In the UHR group: increased WASO, decreased efficiency, increased movements, and number of awakenings were longitudinally associated with symptoms over 12-month follow-up.
Lunsford-Avery (38)	USA	Longitudinal	*N* = 34 UHR (56% females; aged 18.79 ± 1.93 years) + 32 HC	Actigraphy	• UHR individuals displayed significantly more fragmented circadian rhythms and later onset of nocturnal rest compared to HC. • In the UHR group: Circadian disturbances were associated with greater psychotic symptoms at baseline, and predicted severity of symptoms and psychosocial dysfunction at 12-months follow-up.
Mayeli (21)	USA	Cross-sectional	*N* = 22 UHR (54% females; aged 20.3 ± 4.6 years) + 20 HC	• hd-EEG • Polysomnography	• UHR individuals had more WASO and higher NREM sleep gamma EEG power in a large fronto-parieto-occipital area compared to HC. • No significant difference between groups was found regarding arousal index during NREM sleep. • In the UHR group: higher NREM sleep gamma power in medial frontal-anterior frontal and posterior regions was related to worse negative symptoms.
Ristanovic (22)	USA	Cross-sectional	*N* = 57 CHR (aged 18.89 ± 1.82) including 38 participants who had actigraphy data collected + 61 HC	Actigraphy	• Automatic maladaptive responsivity to family stressors (i.e., greater involuntary engagement stress response) was associated with disrupted sleep (i.e., poorer sleep efficiency) in the CHR but not HC group. • Impaired stress tolerance was associated with all objectively assesses sleep parameters (i.e., sleep duration, continuity, and efficiency).
Zanini (2015) (32)	Brazil	Cross-sectional	*N* = 20 UHR (35% females: aged 18.3 ± 3.91) + 20 HC Females of the UHR group=35%	Polysomnography PSQI ESS MEQ	• UHR individuals reported significantly poorer sleep quality than HC (PSQI total scores of 7.70 (±3.68 vs. 4.95 ± 2.16, respectively; *p* = 0.007). • No differences found between groups regarding sleepiness and chronotype profiles. • Polysomography findings indicated that the UHR group presented significantly higher sleep latency onset and REMOL than HC.

^*^UHR state was evidenced using structured interviews (e.g., CAARMS, Comprehensive Assessment of the At Risk Mental State; SIPS, Structured Interview for Prodromal Symptoms; SOPS, Scale of Prodromal Symptoms).

FEP, First Episode Psychosis; HC, Healthy controls; RCT, Randomized Controlled Trial; PSQI, Pittsburgh 464 Sleep Quality Index; ESS, Epworth Sleepiness Scale; MEQ, Morningness and Eveningness Questionnaire; RU-SATED, Regularity, Satisfaction, Alertness, Timing, Efficiency, Duration; hd-EEG, High Density Electroencephalography; WASO, Wakefulness After Sleep Onset; NREM, Non-Rapid Eye Movement; REMOL, Rapid Eye Movement Sleep Onset Latency.

### Subjective sleep disturbances in UHR individuals

Most of the existing studies found that sleep disturbances were highly prevalent in UHR individuals based on the structured interviews SIPS/SOPS sleep disturbance scores (27–30); and were reported to a greater extent by UHR groups compared to clinically lower risk patients (29) and healthy volunteers (30, 31). Beyond general sleep disturbances, some previous studies documented worse overall sleep quality (according to higher PSQI scores) in UHR individuals as compared to healthy controls (12, 20, 32–34), and even to first episode psychosis (FEP) patients (34). In particular, UHR individuals displayed longer sleep latency (i.e., the amount of time between reclining in bed and the onset of sleep) (12, 32, 35), shorter sleep duration in hours per night (20), greater daily sleep disturbances (e.g., night time and early morning awakenings) (12), greater daytime dysfunction due to sleepiness (20, 34), and increased sleep medication use (20, 34). Conversely, other studies did not find any differences in self-reported sleep latency (34) sleep duration (12, 34) and sleepiness scores (33) between UHR subjects and healthy controls. One prior research showed that UHR respondents exhibited significantly higher self-reported nightmare frequencies compared to healthy controls, and more dream recall frequencies compared to patients with schizophrenia (36). Finally, two studies using the Morningness and Eveningness Questionnaire (MEQ) (37) found no significant differences in self-reported chronotype profiles between UHR and healthy individuals (32, 33).

### Objective sleep disturbances in UHR individuals

We could find only two polysomnography studies, which revealed that the UHR group presented significantly more Wake After Sleep Onset (WASO) (21) and increased sleep latency (i.e., more difficulty falling asleep) than HC (32). Zanini et al. (32) did not find significant differences in the polysomnographic sleep efficiency percentages, WASO scores, and total sleep time and of UHR participants relative to controls. The actigraphy data indicated that UHR individuals experienced increased WASO, decreased efficiency (22), increased night time movements (35), longer daytime nap duration (33), more fragmented circadian rhythms (33, 38) and later onset of nocturnal rest (38) compared to healthy controls. Based on sleep high density-Electroencephalography (hd-EEG) recordings, Mayeli et al. (21) found that UHR participants presented increased EEG gamma activity during non-rapid eye movement sleep in a large fronto-parieto-occipital area compared to controls.

### Summary of past subjective and objective studies

In sum, previous studies highlighted a wide range of both subjectively and objectively assessed sleep disturbances in UHR individuals, including increased sleepiness, daytime naps, sleep latency, night time movements, nightmares, and a disrupted circadian rhythm. Overall, studies using subjective measures were more represented in the existing literature than those using objective measures, and mainly focused on general sleep disturbance [non-specific sleep disturbance severity scales of the SIPS/SOPS; *n* = 7 studies (27–31, 39, 40)], sleep quality [SPSQI; *n* = 7 studies (12, 20, 32–35, 41)], sleepiness [ESS; *n* = 2 (32, 33)], and chronotype [MEQ; *n* = 2 studies (32, 33)]. However, yet no studies assessed insomnia symptoms in UHR subjects using self-report measures [e.g., Insomnia Severity Index (42), Athens Insomnia Scale (43)]; despite having shown to be prevalent and severe in patients with psychosis (44, 45). We could identify only six previous studies using objective sleep measures; four of them used Actigraphy (33, 35, 38) and two used polysomnography (21, 32). All these studies involved small sample sizes (20–38 UHR individuals), and four of them had a cross-sectional design. These identified gaps may limit the conclusions drawn from the current review, and contribute to give an insight to future research in this field. Additional long-term cohort observation studies using objective sleep parameters and large sample sizes to better represent the UHR population are still very needed. It is also important that we draw attention to the fact that all studies in this topic have been performed in Western countries. Research from other parts of the world would be highly informative and should be encouraged.

## Associations between sleep disturbances and clinical/functional outcomes in UHR individuals

Sleep disturbances have proven to be associated with various negative outcomes in patients with early and chronic psychosis, including heightened psychotic symptom severity, greater suicidality, increased cognitive deficits, as well as impaired functioning and quality of life (11, 46, 47). In this section, we propose to review the available research on the effects of sleep disturbances on various clinical and functioning outcomes in UHR individuals.

### Associations between sleep disturbances and clinical outcomes

A limited amount of research has specifically addressed the contribution of sleep disturbances to psychotic symptoms in UHR individuals (11, 48–50). Some studies found that several disrupted sleep parameters at baseline (e.g., increased WASO, reduced efficiency, heightened night-time movements and more awakenings, lowered sleep time, fragmented circadian rhythm) were significant longitudinal predictors of the later severity of positive (35, 38, 50) and negative (38) psychotic symptoms. A recent longitudinal study (20) tracked sleep during 8 months in 478 UHR individuals, and showed that sleep disturbance was strongly and prospectively linked to increased psychotic symptoms (positive, negative, disorganized, and general) over time. Lunsford-Avery et al. (35) found that both baseline self-reported and actigraphic-measured sleep disturbance (i.e., decreased sleep efficiency, increased WASO, greater number of awakenings, and increased movements) helped predict the longitudinal course of positive symptoms specifically in UHR individuals; whereas no significant correlation has been found between actigraphic variables and negative symptoms over time. More particularly, findings from a large retrospective study from the UK demonstrated a specific association between sleep problems and greater perceptual abnormality frequency and severity in young UHR individuals (19). Similarly, Goines et al. (31) pointed to the specific effect of hallucinations on sleep over other attenuated psychotic symptoms. While these two studies (19, 31) suggested that perceptual abnormalities may lead to sleep disturbances in UHR individuals, other authors claimed a reversed pattern of association (51); highlighting the need for additional studies to elucidate the direction and nature of this relationship.

To summarize, the majority of the existing longitudinal studies cited above agreed that sleep disturbances are associated with subsequent psychotic symptoms exacerbation, which may in turn lead to poor mental health and functional outcomes even in individuals recovering from their at-risk state (52, 53). However, the observed impact of sleep disturbance on psychotic symptoms evolution over time is still unclear mainly due to a lack of longitudinal studies (20), which highlights the need for further investigations.

### Associations between sleep disturbances and functional outcomes

Sleep problems have been consistently shown to impact cognitive processes and daytime functioning (54). Severe cognitive deficits and subsequent daily functional impairment are common outcomes in UHR youth, regardless of eventual conversion to threshold psychosis (53, 55, 56). Some cross-sectional evidence suggested that sleep disturbances are associated with more psychosocial dysfunctioning (30, 41). However, to date, little attention has been paid to the prospective relationship between sleep and functional outcomes in UHR individuals (10). One longitudinal research by Lunsford-Avery et al. found that objectively assessed circadian rhythm disruption at baseline predicted worse psychosocial functioning levels at 12-months follow up (38). More recently, Nuzum et al. (19) investigated 795 clinical records of UHR patients and found that sleep problems predicted worse follow-up levels of social functioning. Although sleep is a potentially modifiable factor (57, 58), that when treated could substantially and independently improve functional outcomes (59), its impact on functioning, quality of life and overall wellbeing in UHR states remain largely understudied (10). Therefore, further studies allowing a deeper understanding of the pathways linking sleep to developmental and long-term outcomes in UHR youth are required.

## Sleep disturbance as a risk factor for transition to psychosis

It is well-established that a proportion of UHR individuals will convert later to a clinical psychosis (60, 61). Although evidence showing that sleep disturbances are present in UHR individuals before any psychosis onset (8, 31, 32, 39), there has been very limited interest so far on how abnormal sleep patterns may contribute to the transition risk (20). The first attempt to use sleep disturbances in the prediction of transition to psychosis was performed by Ruhrmann et al. (39) through a large prospective European study (39), and documented sleep as a strong predictive factor of transition from subthreshold to full-threshold psychosis over 18 months of follow-up (39). Inconsistent findings have emerged thereafter; with some studies showing that sleep problems represented a risk indicator for both the persistence and/or exacerbation of psychotic symptoms over time (35, 62); while others found no significant effect of sleep on psychosis transition (20, 30, 31). More recently, Nuzum et al. (19) found that, despite a lack of significant association between sleep parameters and transition to psychosis, sleep disturbances were significantly correlated with a shorter time to transition in UHR individuals converted to psychosis. Zaks et al. (20) suggested that disrupted sleep patterns may act as risk factors for transition only in aggregation with other potential factors. Overall, the pathways between sleep and psychosis are rather complex and have been hypothesized to be bidirectional (63). The eventual role that sleep would play in the development and progress of psychotic symptoms raises the question of its underlying mechanisms of the effects of sleep on the pathogenesis of psychosis (11, 49, 64). We propose to address this question in the next section.

## Explaining mechanisms of the relation sleep disturbances–psychosis

The implication of sleep on the development and progress of psychosis has attracted a growing interest during the last years. A key hypothesis that has particularly received attention is the presence of structural brain abnormalities and neural development alterations which may likely lead to sleep alterations, and in turn psychotic symptoms, from the very early stages of psychosis (64). Structural brain alterations in the thalamic region are involved in both human sleep dysregulation (65), and the etiopathogeny of schizophrenia (66, 67). A thalamic volume reduction is noted early during the course of psychotic disorders, and exacerbates as the disease progresses (68). In this regard, a study by Lunsford-Avery et al. revealed that UHR individuals had a reduced bilateral thalamic volume that significantly related to poorer sleep quality (12). A recent review suggested that aberrant thalamic function could result in sleep spindle deficits and altered EEG microstate dynamics (69). These alterations [e.g., increased non-rapid eye movement gamma EEG activity (21)] have been previously reported in UHR individuals, and could, in turn, represent “potent endophenotypes” and “vulnerability factors” for psychosis (69). This brings us back to the hypothesis stated earlier by Feinberg (70), that the great rearrangement of brain function and structure occurring during early adolescence, including maturational changes in EEG sleep patterns (i.e., decline in EEG amplitude), may have resulted from a decrease in cortical synaptic density, which may in turn underlie the emergence of schizophrenia. In this line of research, a relevant hypothesized biological mechanism underlying the effect of sleep on psychosis risk is a defective synaptic transmission. Acetylcholine has been shown to play a major role in both sleep and hallucinatory phenomena (71). As such, it has been suggested that sleep deprivation can be a direct cause of hallucinations and progression toward psychosis due to cholinergic depletion (51).

Other explaining mechanisms for the association between sleep and subsequent psychosis have been identified, such as negative affects (i.e., depression and anxiety) (72–74), endocrine dysfunction due to exposure to psychosocial and biological stress, and cognitive deficits (64). In this regard, some biological markers have been found to interfere with both psychosis proneness and sleep disturbances through a stress-induced activation of the hypothalamic–pituitary–adrenal (HPA) axis, including elevated cortisol levels (75, 76) and pro-inflammatory cytokines (77, 78). Interestingly, an increased reactivity to stress has recently been found to directly affect sleep in the UHR stage. For instance, Ristanovic et al. (22) found that maladaptive responsivity to family stressors and impaired stress tolerance were associated with disrupted sleep as assessed objectively in UHR subjects. Another previous study (41) suggested a pathway in which sleep problems amplify stress indirectly through a negative effect on cognition.

All the above-mentioned factors would interplay and together contribute to sleep disturbances and psychosis in vulnerable young people, highlighting “a possible role for sleep dysfunction within a neurodevelopmental diathesis–stress model for schizophrenia” (64). In sum, despite increased efforts to elucidate the complex relationship sleep-psychosis, a long way remains ahead before understanding what mechanisms are behind the observed patterns of relationships between these two constructs.

## Prevention and intervention strategies targeting sleep for UHR individuals

Our review of the literature identified several altered subjective and objective sleep parameters in the at-risk stage of psychosis (10, 16), that have even been hypothesized as causal mechanisms of psychosis development and persistence (49, 79). Sleep is a malleable factor that has been shown to worsen psychotic symptoms when non-treated (20, 35, 62, 72), or reduce them and improve functional outcomes when treated (59, 80). As such, we suggest that although the directionality and the specific mechanisms underlying the relationship sleep-psychosis have not yet been elucidated, treatment of sleep problems has the potential to either reduce the risk of developing psychosis (as a causal factor) (49, 79), or prevent worsening of the disease outcomes (as an effect of prodromal symptoms) (59, 80).

When present, sleep disturbances are recommended to be screened for, monitored and treated according to the DSM-5 recommendations (81). Therefore, we suggest that screening for and addressing sleep disturbances should be a routine practice in early intervention services to help with reducing their clinical and functional impact on young UHR individuals (82). Promoting sleep in this vulnerable population may be a promising intervention target for symptom and functioning improvement (20), and possibly for delay or prevention of transition to psychosis (39, 83).

Sleep management in psychosis has been shown to be limited by several challenges. First, sleep disturbances are often not given the required focus (10) and are thus underestimated and undertreated. Second, clinicians tend to evaluate sleep informally and offer no or inadequate treatment (e.g., pharmacotherapy instead of the recommended first-line psychotherapy) (84, 85). Third, at the present time no consensual recommendations are available for the management of sleep problems in UHR patients. Basic sleep hygiene education has proven to be beneficial in people with psychosis (11, 46). In this regard, some authors demonstrated the benefits of a low cost and simple sleep intervention for early psychosis patients, consisting of sleep hygiene advice along with a provision of a wearable sleep tracker (86). Accumulated evidence has confirmed the effectiveness of the Cognitive Behavioral Therapy for insomnia (CBTi) in UHR states (80, 82, 87). The CBTi has also been proven to have wider benefits by improving depression, anxiety (44, 80, 88), and attenuated psychotic symptoms (44, 80). Other sleep interventions have been specifically designed and tested for use in young UHR people, such as the brief psychological intervention “SleepWell” that targets key sleep parameters (i.e., hyperarousal, sleep pressure, and circadian rhythm), and has yielded promising preliminary results (82). The SleepWell is now being tested in a randomized trial in young patients at ultra-high-risk of psychosis [for further details, see the published protocol (89)].

## Conclusion and perspectives

The current review extends our existing knowledge by further highlighting the presence of disturbed sleep in UHR individuals as evidenced by subjective and objective sleep measures such as polysomnography, sleep electroencephalograms, and actigraphy. Reviewing the possible mechanisms underlying the relationship between sleep and psychosis emphasizes its complex and multifactorial nature which is yet to be determined and understood. Our literature search revealed that this topic has attracted relatively scant research attention so far. Nevertheless, the early intervention field is a growing area of research and we expect that studies on sleep and UHR will continue to increase during the coming years. As such, we believe that our Mini review will shed light on the importance of further focusing on this promising avenue of research. Further high-quality longitudinal and experimental research on sleep involving large samples of UHR individuals and using a broad range of sleep parameters is warranted to determine which facets of sleep disturbances are most detrimental to this specific population, and to what extent they can be causal factors or markers of psychosis. This research can help deepen our understanding of the continuum of psychosis vulnerability; and inform psychosis-risk prediction models as well as prevention and early intervention programs.

## Author contributions

FF-R wrote the first draft. SH, MC, and HJ provided intellectual contributions to strengthening the manuscript. All authors were involved in revising the manuscript and approved the final version.

## Conflict of interest

The authors declare that the research was conducted in the absence of any commercial or financial relationships that could be construed as a potential conflict of interest.

## Publisher's note

All claims expressed in this article are solely those of the authors and do not necessarily represent those of their affiliated organizations, or those of the publisher, the editors and the reviewers. Any product that may be evaluated in this article, or claim that may be made by its manufacturer, is not guaranteed or endorsed by the publisher.
